# The effect of cytokines produced by human adipose tissue on monocyte adhesion to the endothelium

**DOI:** 10.1080/19336918.2019.1644856

**Published:** 2019-07-22

**Authors:** Sona Cejkova, Hana Kubatova, Filip Thieme, Libor Janousek, Jiri Fronek, Rudolf Poledne, Ivana Kralova Lesna

**Affiliations:** a Laboratory for Atherosclerosis Research, Institute for Clinical and Experimental Medicine, Prague, Czech Republic; b Department of Physiology, Faculty of Science, Charles University, Prague, Czech Republic; c Center for Experimental Medicine, Department of Transplant Surgery, Institute for Clinical and Experimental Medicine, Czech Republic; d Department of Anesthesia and Intensive Medicine, First Medical Faculty, Charles University and University Military Hospital, Czech Republic

**Keywords:** Atherosclerosis, cytokines, inflammation, endothelium, adipose tissue

## Abstract

Visceral adipose tissue (VAT) may play a critical role in atherosclerotic cardiovascular disease. The goal of this study was to determine the effect of human VAT-released pro‑inflammatory cytokines on monocyte adhesion to the endothelium. The cytokine effects on monocyte adhesion to the endothelial cells (ECs) were tested using adipose tissue-conditioned media (ATCM) prepared by culturing human VAT. The cytokines concentrations in ATCM, the cytokines expression and adhesion molecules in stimulated ECs were measured. The concentrations of IL-1β,TNF-α,MCP-1,IL-10,and RANTES measured in ATCM correlated positively with monocyte adhesiveness to ECs. Additionally, ATCM increased the adhesion molecules (ICAM-1, VCAM-1) gene expression. Selective inhibitors highlighted the importance of IL-1β and TNF-α in the process by a significant decrease in monocyte adhesion compared to ATCM preconditioning without inhibitors. Human VAT significantly increased monocyte adhesion to ECs. It was significantly influenced by IL-1β, TNF-α, MCP-1, IL-10, and RANTES, with IL-1β and TNF‑α having the strongest impact.

## Introduction

The driving factor in the development of atherosclerosis and its clinical complications could be subclinical inflammation of arteries and their surrounding structures. Based on previous studies, one of the main players in this process could be adipose tissue [1,2]. Therefore, a better understanding of the mechanisms that underlie the role of adipose tissue in vascular disease, that is, atherosclerosis, could be of key importance, especially when faced with the increasing prevalence of obesity worldwide. It has been already shown that the inflammatory processes provoked by modified low-density lipoprotein (LDL) particles in the vessel wall pathologically activate the endothelium and could lead to serious arterial dysfunction and even occlusion caused by the atherosclerotic process [3–6]. The key players in these processes are monocytes entering the subintimal space and activated to macrophages. However, the exact mechanisms of monocyte activation are still not fully understood. One of the main mediators could be pro-inflammatory cytokines, which lead to monocyte rolling, adhesion and extravasation from the bloodstream into lesion-prone areas of the arterial vasculature [7]. In this process, adipose tissue, its content and the activity of monocytes/macrophages could be of utmost importance. In this respect, visceral adipose tissue (VAT) seems to be the most dangerous [8,9]. Indeed, in our previous studies, we found substantially higher gene expression of pro-inflammatory cytokines and higher infiltration of pro-inflammatory, polarized macrophages in human VAT than in subcutaneous adipose tissue [10]. In this study, to further evaluate the role of pro-inflammatory cytokines produced by VAT, we analyzed their effect on monocyte adhesion to the endothelium.

The aims of the present study were to identify the VAT-released cytokines which could potentially affect the human endothelium and monocyte adhesiveness *in vitro* and, also, to identify the cell type being the main source of pro-inflammatory cytokines by comparing of adipose tissue-conditioned media (ATCM) and stromal vascular fraction-conditioned media (SVFCM). The study is unique in describing the effect of human adipose tissue-released products on the initial stages of atherosclerosis.

## Results

The basic parameters obtained in the group of studied individuals such as age, smoking status, body mass index (BMI), plasma total cholesterol and C-reactive protein measured by a highly sensitive method (hs-CRP) were similar in men and women (Table 1). BMI was lower and the prevalence of smokers higher than in a 1% representative sample of the Czech population [11].

**Table 1.** Basic physiological and clinical characteristics of the analysed groups of living kidney donors shown as mean ± SD.

	Total (n = 30)	Men (n = 11)	Women (n = 19)
Age (years)	46.2 ± 12.1	44.7 ± 4.6	47.1 ± 10.5
Body mass index (kg/m^2^)	26.1 ± 4.20	27.5 ± 3.38	25.2 ± 4.14
Smoking (%)	46.7	45.5	47.4
Total cholesterol (mmol/L)	5.41 ± 1.52	5.18 ± 2.47	5.57 ± 1.70
hsCRP (mg/L)	1.88 ± 2.36	1.28 ± 0.45	2.43 ± 3.15

### Effects of ATCM on monocyte adhesion to the endothelium

**Figure 1.**
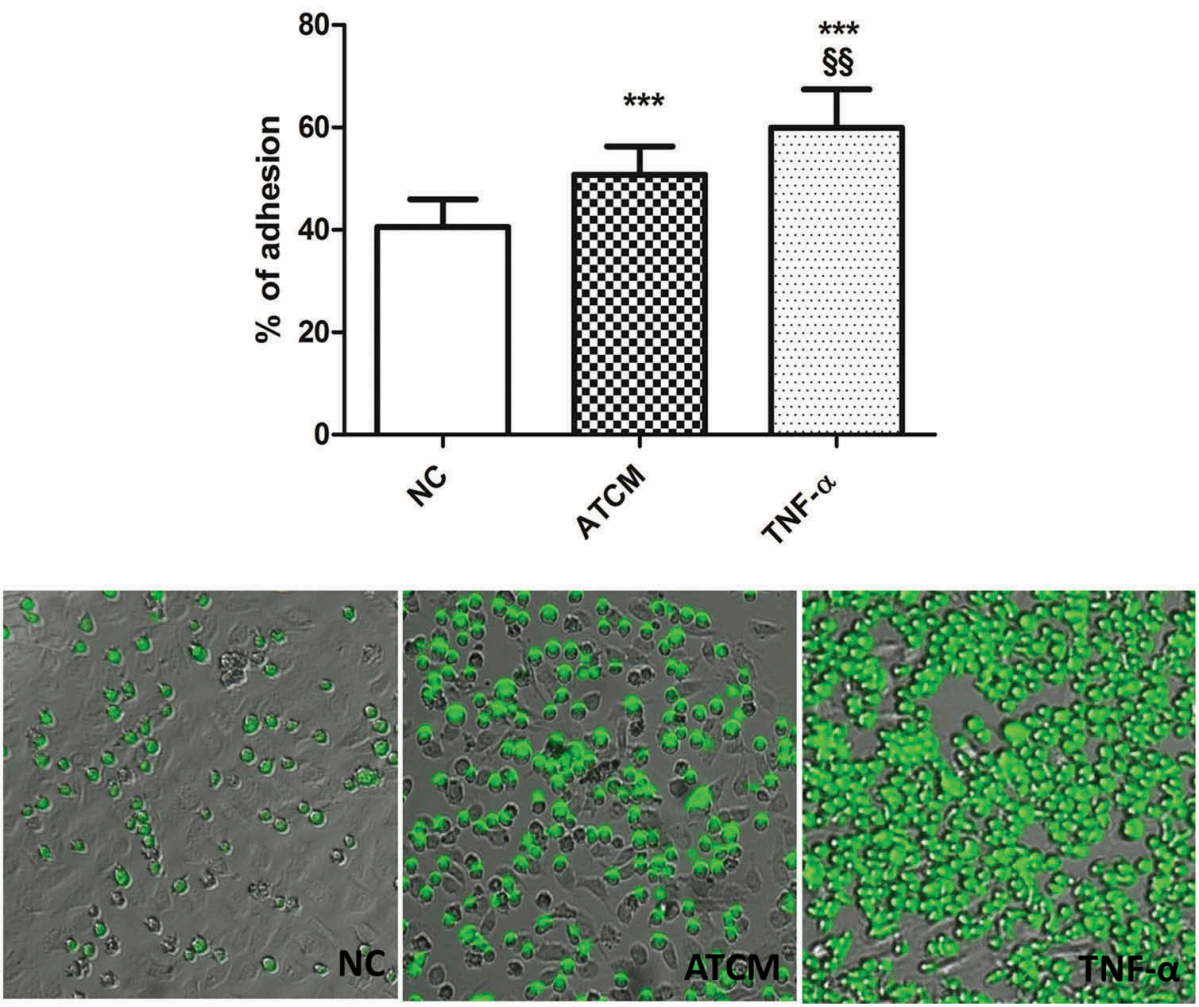
ATCM significantly increased (p < 0.0001) the level of cell adhesion compared to the negative control (NC). TNF-α (10ng/ml) used as a positive control significantly increased the percentage of adhesion of HUVECs. *** p < 0.0001 compared to NC; §§ p < 0.001 compared to positive control, NC – negative control, ATCM – adipose tissue-conditioned media, TNF-α – tumor necrosis factor α.

The first main finding of our study was that preconditioning of human umbilical vein endothelial cells (HUVECs) using ATCM increased monocyte adhesion to the endothelium by a quarter compared to the control medium (50.8% ± 5.6 ATCM vs. 40.6% ± 5.4 negative control, respectively; p < 0.0001). At the same time, after the addition of tumor necrosis factor-α (TNF-α, 10 ng/ml) to the culture medium, adhesion of monocytes using ATCM was lower than in positive controls (60.0% ± 7.5, p < 0.0001) (Figure 1)

**Figure 2.**
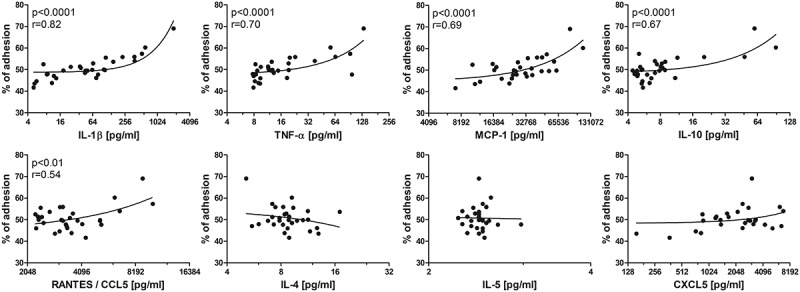
Correlation of the level of adhesion to cytokine concentrations in ATCM. The X-axis has a log_2_ scale. IL-1β/4/5/10 – interleukin 1β/4/5/10, TNF-α – tumour necrosis factor α, MCP-1 – monocyte chemoattractant protein-1 (CCL2), RANTES – CCL5 – regulated the activation, normal T cell expression and secretion.

The second main finding was that ATCM also increased monocyte adhesion proportionally to the concentrations of IL-1ß (IL-1ß, p < 0.0001, r = 0.82), TNF-α (p < 0.0001, r = 0.70), monocyte chemoattractant protein 1 (MCP-1, p < 0.0001, r = 0.69), and RANTES (CXCL5, p < 0.01, r = 0.54) (Figure 2). Quite unexpectedly, monocyte adhesion correlated positively with IL-10 concentration (p < 0.0001, r = 0.67). Finally, no correlation between monocyte adhesion and IL-4, IL-5 or CXCL5 concentrations was found.

**Figure 3.**
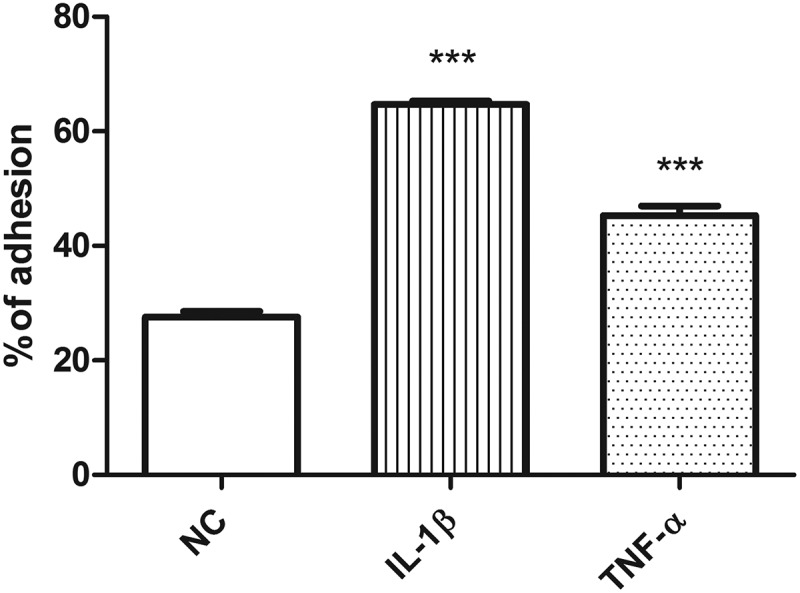
Stimulation of endothelial cells by IL-1ß and TNF-α at concentrations measured in ATCM. IL-1ß (200 pg/ml) and TNF-α (30 pg/ml). NC – negative control, IL-1ß – interleukin 1ß, TNF-α – tumor necrosis factor α, * p < 0.05, *** p < 0.001.

In addition, stimulation of endothelial cells (ECs) by IL-1ß (200 pg/ml) and TNF-α (30 pg/ml) at concentrations much lower than usually used in *in vitro* models using ATCM significantly increased monocyte adhesion to the endothelium (Figure 3).

### Gene expression in endothelial cells influenced by ATCM

**Figure 4.**
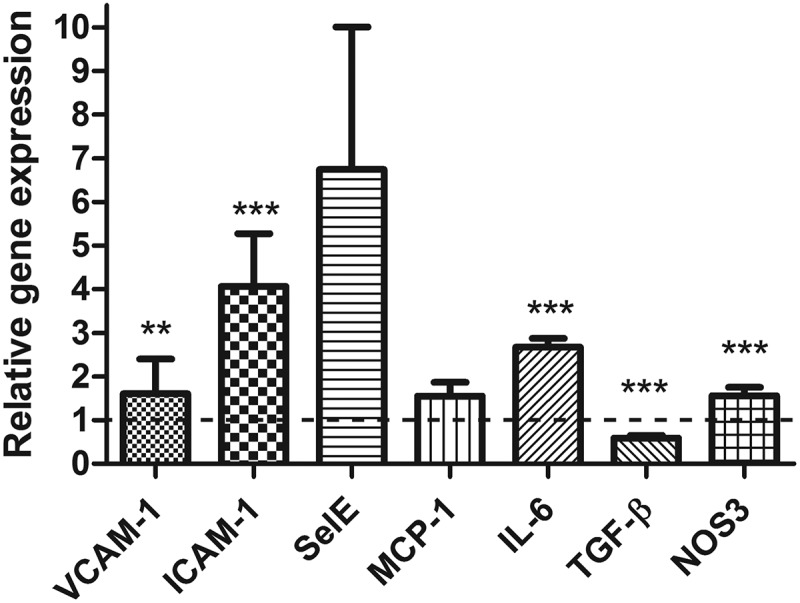
Effect of ATCM on relative gene expression in endothelial cells compared to the negative control. ** p < 0.01, *** p < 0.001, SelE p = 0.089. ICAM-1 – intercellular adhesion molecule 1, VCAM-1 – vascular cell adhesion molecule 1, SelE – selectin E, MCP-1 – monocyte chemoattractant protein-1 (CCL2), IL-6 – interleukin 6, TGF-β – transforming growth factor β, NOS3 – nitric oxide synthase type 3

Preconditioning of ECs by ATCM significantly increased gene expression of the adhesion molecules vascular cell adhesion protein 1 (VCAM-1, 1.60-fold, p < 0.01) and intercellular cell adhesion protein 1 (ICAM-1, 4.07-fold, p < 0.01), which are important for the adhesion process (Figure 4). Moreover, ATCM increased pro-inflammatory IL-6 (2.64-fold, p < 0.0001) and decreased transforming growth factor β (TGF-β, 0.59-fold, p < 0.0001) gene expression in ECs more than in the negative control. The gene expression of MCP-1 and selectin E (SelE) was not significantly influenced in the whole group due to the high individual differences in the case of the SelE molecule.

### Effect of selective cytokine inhibition

To evaluate the real role of individual cytokines in the process of endothelial activation and monocyte adhesion to the endothelium, selective cytokine inhibitors were used.

**Figure 5.**
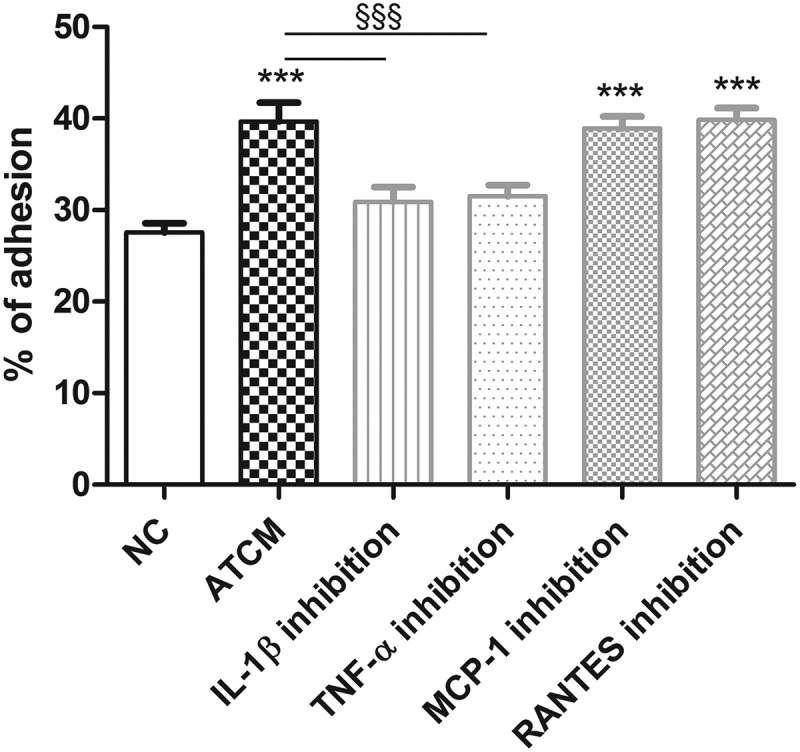
Effect of inhibition of selected cytokines during stimulation of endothelial cells. NC – negative control, ATCM – adipose tissue-conditioned media, IL-1ß – interleukin 1ß, TNF-α – tumor necrosis factor α, MCP-1 – monocyte chemoattractant protein-1 (CCL2), RANTES – CCL5 – regulated the activation, normal T cell expression and secretion, *** p < 0.001 compared to NC, §§§ p < 0.001 compared to ATCM stimulation.

Inhibition of IL-1ß and TNF-α significantly decreased monocyte adhesion ACTM-activated ECs whereas no effects of MCP-1 and RANTES inhibitors were demonstrated (Figure 5).

**Figure 6.**
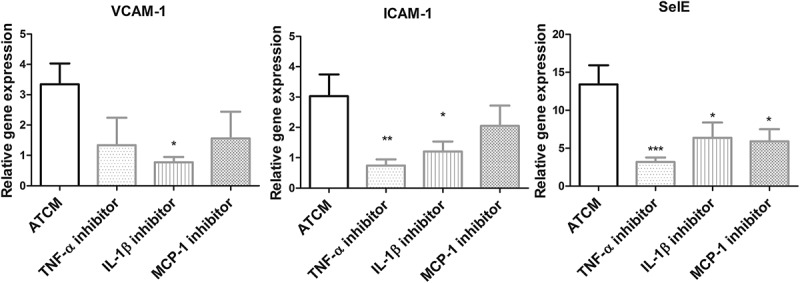
Relative gene expression of adhesion molecules with the addition of TNF-α, IL-1ß and MCP-1 inhibitor compared to negative control. Inhibition of selected cytokines decreased gene expression of adhesion molecules – VCAM-1, ICAM-1 and SelE compared to negative control (unstimulated endothelial cells). VCAM-1 – vascular cell adhesion molecule 1; ICAM-1 – intercellular adhesion molecule 1; SelE – selectin E; IL-1ß – interleukin 1ß; TNF α – tumor necrosis factor α; MCP-1 – monocyte chemoattractant protein-1 (CCL2), * p < 0.05, ** p < 0.01, *** p < 0.0001.

The increase in adhesion molecules (VCAM-1, ICAM-1, and SelE) induced by ATCM stimulation was partially abolished by selective IL-1ß, TNF-α and MCP-1 inhibitors. The effect shown to be the strongest was that on SelE gene expression, as all inhibitors significantly decreased its gene expression with the most prominent effect being that of the TNF-α inhibitor (3.17 ± 0.71 vs. 13.4 ± 2.52, p < 0.001) (Figure 6). TNF-α highly significantly decreased ICAM-1 gene expression (1.34 ± 0.91 vs. 3.34 ± 0.68, p < 0.01) and a minor, but also significant, the effect of IL-1ß was shown (1.21 ± 0.33 vs. 3.03 ± 0.71, p < 0.05). Gene expression of VCAM-1 was significantly decreased when IL-1ß had been inhibited (0.77 ± 0.18 vs. 3.34 ± 0.68, p < 0.05); while TNF-α (p = 0.09) and MCP-1 (p = 0.19) inhibition did not reach the level of significance, a trend could be clearly seen.

### Comparison of ATCM and SVFCM in cytokine production

When comparing SVFCM and ATCM, no difference was found in IL-1β level, and a significantly higher concentration was found for TNF-α (Table 2). By contrast, the concentrations of IL-6, MCP-1, RANTES, and IL-10 were significantly higher in ATCM than in SVFCM. The IL-6, IL-10, MCP-1, and RANTES concentrations in ATCM were significantly higher than those in SVFCM for the same subject.

**Table 2.** Comparison of cytokine concentrations in ATCM and SVFCM shown as mean ± SD. IL-1β/6/10 – interleukin 1β/6/10; TNF-α – tumour necrosis factor α; MCP-1 – monocyte chemoattractant protein-1 (CCL2); RANTES – regulated the activation, normal T cell expression and secretion (CCL5).

	ATCM	SVFCM	p value
IL-1β (pg/mL)	605.8 ± 148.7	699.4 ± 124.3	0.7072
TNF-α (pg/mL)	266.8 ± 33.00	695.7 ± 125.1	0.0159
IL-10 (pg/mL)	127.2 ± 10.4	84.10 ± 5.73	0.0008
IL-6 (ng/mL)	142.8 ± 21.87	16.71 ± 3.31	0.0007
MCP-1 (ng/mL)	39.21 ± 5.26	8.40 ± 1.65	0.0006
RANTES (ng/mL)	4.23 ± 0.66	2.003 ± 0.09	0.0079

### Potential impact of extreme values

Among all genes being investigated, the largest difference between the highest and lowest quartiles was found for the expression of VCAM-1 (4.36 ± 3.1 vs. 1.01 ± 0.8, p = 0.011) (Table 3). On the one hand, no significant difference in the mean values of MCP-1 expression between ATCM and negative control was found. On the other hand, we observed a significant difference between the highest and lowest quartiles (3.19 ± 2.6 vs. 0.80 ± 0.04, p = 0.022). No similar relationships were found for other genes. This is consistent with the positive correlation between the gene expression of VCAM-1 (p < 0.05, r = 0.44) and MCP-1 (p < 0.05, r = 0.38) involved in monocyte adhesion to ECs. No differences between the means were noted, and interquartile comparisons for SelE gene expression were between the ATCM and negative control groups despite the highest mean value due to variations in individual values. Nevertheless, our analysis of the whole group showed a significant correlation between the relative gene expression of SelE and monocyte adhesion (p < 0.0001, r = 0.73). The differences between ATCM and negative control observed in the extreme quartiles of TGF-β reached borderline significance (0.40 ± 0.9 vs. 0.60 ± 0.2, p = 0.063). Although an increased relative gene expression of ICAM-1 and IL-6 in ATCM-treated ECs was documented, no significant differences were observed between their extreme quartiles and monocyte adhesion.

**Table 3.** Comparisons of gene expression analysis of the level of monocyte adhesion in the extreme quartiles. VCAM-1 – vascular cell adhesion molecule 1; ICAM-1 – intercellular adhesion molecule-1; SelE – selectin E; TGF-β – transforming growth factor β; IL-6 – interleukin 6; MCP-1 – monocyte chemoattractant protein-1 (CCL2); NOS3 – nitric oxide synthase type 3.

	Upper quartile	Lower quartile	p value
VCAM-1	4.36 ± 3.1	1.01 ± 0.8	0.011
SelE	19.45 ± 29.5	0.33 ± 0.2	0.088
ICAM-1	7.89 ± 10.7	1.37 ± 0.83	0.107
TGF-β	0.60 ± 0.2	0.40 ± 0.2	0.063
IL-6	2.00 ± 1.1	2.84 ± 1.0	0.130
MCP-1	3.19 ± 2.6	0.80 ± 0.04	0.022
NOS3	1.22 ± 0.3	1.34 ± 0.6	0.619

When including cardiovascular risk factors (sex, age, menopause, BMI, smoking, hs-CRP concentration) in the analyses, no effect on the level of monocyte adhesion to ECs was shown. However, when comparing the extreme quartiles of the level of monocyte adhesion to ECs, we observed a moderately lower mean BMI than in the highest quartile (25.8 ± 2.96 kg/m^2^ vs. 28.9 ± 2.97 kg/m^2^, p = 0.064). An opposite relationship was found for age (lower extreme quartile 51.1 ± 10.5 years vs. the upper extreme quartile 42.9 ± 13.7, p = 0.198). However, this finding may have been influenced by the relatively small number of subjects included in the study.

## Discussion

Our results show that human VAT-released cytokines directly increase monocyte adhesion *in vitro*. The level of monocyte adhesion positively correlates with the concentrations of IL-1β, TNF-α, MCP-1, RANTES and IL-10 measured in ATCM. These results imply that human VAT is an important source of pro-inflammatory cytokines, which possibly affects local inflammation within adipose tissue as well as systemic low-grade inflammation. In addition, we confirmed that it is the cells of stromal vascular fraction (SVF) that are the main source of pro-inflammatory cytokines.

This finding is consistent with the previously reported pro-atherogenic effects of pro-inflammatory cytokines in experimental models [12–15]. These effects have also been demonstrated to play an important role in the development of obesity-related diseases [16–20]. Our results are in agreement with the work of Henrichot et al. [15] focusing on the effect of ATCM on chemotaxis and effect of IL-8 and MCP-1. However, according to our results, the effect of MCP-1 on cell adhesion was not that strong as shown for chemotaxis [15]. These relationships between cytokines and monocyte adhesion to the endothelium were also found in our human study with a group of normal weight individuals – healthy LKDs.

The unexpected positive correlation between IL-10 concentration and monocyte adhesion could be explained by the synergy of IL-10 with another component of ATCM. This is supported by our finding that, when assessing separately the effect of IL-10 on monocyte adhesion using an identical model, it tended to increase the level of adhesion only moderately (data not presented). However, IL-10 definitely did not decrease the level of adhesion, as reported in an earlier study by Mostafa Mtairag et al. [21]. Data from other studies also indicate that while IL-10 is generally considered an anti-inflammatory cytokine, it decreases EC adhesion in experimental models [21,22]; there is also evidence that IL-10 might have a negative influence on the endothelium [23].

ATCM precondition led to pro-inflammatory changes in EC gene expression *in vitro*. We focused on the adhesive molecules VCAM-1, ICAM-1, and SelE, which play important roles, especially in the initial steps of leukocyte adhesion to the endothelium and are mostly expressed after a pro-inflammatory stimulus [24]. ATCM significantly increased ICAM-1 and VCAM-1 gene expression. Our data on the effect of these adipose tissue-produced cytokines on gene expression are in agreement with their important role in the adhesion process [25].

In addition to changes in the gene expression of adhesion molecules, which affect monocyte behavior, ATCM significantly increased the expression of pro-inflammatory IL-6 and nitric oxide synthase (NOS3), thereby affecting EC behavior. In this setting, the production of molecules increased the adhesiveness and, additionally, also that of pro-inflammatory molecules in ECs. These two effects act synergistically. At the same time, the decreased gene expression of anti-inflammatory TGF-β [26–29] potentiated pro-inflammatory changes in ECs in our study. These data documenting a combination of endothelial activation and increased production of adhesive molecules are in agreement with previous studies [29–31]. Moreover, the increased expression of IL-6 in ECs might locally influence macrophage polarization towards pro-atherogenic macrophages [32] in synergy with other pro-inflammatory cytokines (IL-1β and TNF-α) produced mainly by macrophages infiltrated into VAT [33,34]. This process may directly contribute to the development of atherosclerosis by increasing the number of monocytes trapped within the vessel wall, similar to the process of macrophage infiltration into adipose tissue.

To analyze the importance of all the cytokines studied, we inhibited ATCM adhesion by selective inhibition of IL-1β, TNF-α, MCP-1 and RANTES. Inhibition of IL-1β and TNF-α significantly decreased monocyte adhesion compared to stimulation by ATCM alone, whereas no effect of inhibition of MCP-1 and RANTES was shown suggesting a major role of IL-1β and TNF-α released from VAT in this process. When inhibiting IL-1β, TNF-α and MCP-1, the relative gene expression of the adhesion molecules in ECs decreased compared to stimulation by ATCM.

To document that even low concentrations of cytokines in ATCM are capable of changing the level of adhesion, a separate experiment using mean IL‑1β and TNF-α concentrations in ATCM was performed. This control study showed that IL‑1β and TNF-α, at concentrations as low as those in ATCM, significantly increased monocyte adhesion.

When comparing the cytokine concentrations in ATCM and SVFCM from the same adipose tissue in a separate experiment with eight subjects, the IL-6, IL-10, MCP-1 and RANTES concentrations in ATCM were much higher than in SVFCM. The implication is that these four cytokines are produced predominantly by adipocytes. Similar production of IL-1β and only a slightly higher concentration of TNF-α were found in SVFCM suggesting that these cytokines are produced mainly by SVF. In agreement with earlier data [34–37], the main producers might be macrophages. The possibility of cytokine diffusion from adipose tissue must be considered whereas the concentrations of cytokines after SVFCM incubation represent their production in these isolated cells. It supports our opinion that these two cytokines play the most important role in monocyte adhesion. They also elicit the closest relationship between their concentrations and macrophage adhesion when analyzing the effect of adhesion of individual ATCM. In addition, the adhesion and cytokine concentrations of IL-1β and TNF-α in SVFCM alone correlated significantly with monocyte adhesion despite the low number of samples, whereas no relation was found for the concentrations of MCP-1, RANTES and IL-6.

When studying a possible effect of cardiovascular risk factors (age, sex, BMI, smoking and plasma hs-CRP concentration) on monocyte adhesion, none of these parameters was effective. Strictly speaking, our data did not demonstrate any relation of ATCM-influenced adhesion to sex, age, smoking and pro-inflammatory status. Only when comparing two extreme quartiles of the level of adhesion did the difference in BMI reach borderline significance (p = 0.063). One might speculate that the relative changes in BMI in our healthy subjects were not strong enough (16.9–33.3) to detect a significant effect of adipose tissue in our model. The same applies to all other risk factors. This is in agreement with our previous results, as we also did not find an effect of BMI on macrophage polarization in VAT [38] and pro-inflammatory gene expression in VAT [39] in the LKD group.

In conclusion, we have documented that cytokines released from human VAT increase monocyte adhesion to the endothelium and lead to pro-inflammatory changes detected as pro-inflammatory gene expression in preconditioned ECs. Furthermore, we have demonstrated that the most potent pro-inflammatory cytokines in this respect were IL-1β and TNF-α.

## Patients and methods

### Donors of adipose tissue

Visceral adipose tissue was obtained intraoperatively from LKDs during kidney transplantation (from the area outside Gerota’s fascia). All healthy LKDs were informed about the study in detail, and they signed informed consent forms prior to inclusion in the study. The study was approved by the Institute’s Ethics Committee.

### Conditioned media

Adipose tissue was transferred to the laboratory within 15 min, and all visible blood vessels and fibrous tissue were removed with it to be subsequently minced into small pieces (approx. 2 mm^3^); the remaining traces of blood were washed out by PBS (Dulbecco’s Phosphate Buffered Saline, w/o Calcium, w/o Magnesium, Biosera, Nuaille, France).

### Adipose tissue-conditioned media (ATCM)

Adipose tissue-conditioned media (ATCM) were prepared by incubation of cleansed and minced VAT (n = 30) in culture media (1 ml per 1 g of adipose tissue) (EBM-2, Lonza, with 0.2% fatty acid-free bovine serum albumin, Sigma Aldrich, A1595, SLBP3641V, St. Louis, MO, USA) in a thermostat for 24 hours (5% CO_2_, 37°C). The ACTM were separated from tissue using a 150 µm strainer, followed by a 22 µm syringe filter, and kept in a ‒80°C freezer until use.

### Stromal vascular fraction-conditioned media (SVFCM)

The stromal vascular fractions (SVFs) of eight VAT samples were isolated for the preparation of SVFCM to be compared to ATCM. Approximately 2 g of the VAT cleaned from all visible blood vessels and fibrous tissue was exposed to collagenase (c = 0.002 g/ml, Sigma-Aldrich), and then repeatedly filtered and purified. SVFCM were prepared in a fashion similar to ATCM (incubated in 1 ml of a culture medium per SVF isolated from 1 g of VAT).

### Model of monocyte adhesion to the endothelium

The level of monocyte adhesion was evaluated based on the differences in fluorescence intensity, reported as percentages, according to Bae et al. [40]. In particular, fully grown HUVECs (Pooled, Lonza, lot. 369,948) in a 96-well plate were incubated in hexaplicate with 5% ATCM or SVFCM in a culture medium (EBM-2 with 2% FBS) (EBM-2, Lonza) for 24 hours. Adhesion data using conditioned media stimulation were compared to a negative control without the addition of conditioned media. In a separate experiment, selective inhibitors were used to evaluate the role of the individual cytokines released from VAT (AF 12,198 – IL-1β inhibitor, c = 1µM, Cat. No. 1793; C 87 TNF-α inhibitor, c = 10µM, Cat. No. 5484; BMS CCR2 22 – MCP-1 inhibitor, Cat. No. 3129, c = 1µM; DAPTA – RANTES inhibitor, c = 10 nM, Cat. No. 2423; Tocris, R&D Systems). Subsequently, calcein acetoxymethyl (calcein-AM, Molecular Probes)-labelled monocytes (THP-1, 10^5^ cells/well) were added for 30 mins and allowed to adhere to the EC monolayer. After measuring baseline fluorescence intensity, non-adhering monocytes were washed out with PBS three times, with final fluorescence intensity measured using a microplate reader. TNF-α (10 ng/ml, R&D Systems) was used as a positive control.

THP-1 (ECACC 88,081,201) cells were cultured in RPMI-1640 () with 10% fetal bovine serum (FBS) (Gibco™, Thermo Fisher Scientific), 2 mM L-glutamine (Sigma Aldrich) and penicillin-streptomycin (Sigma Aldrich). Human umbilical vein endothelial cells (HUVECs) were cultured using an EGM-2 BulletKit (CC-3156 & CC-4176, Lonza).

### Measurements of the cytokine ATCM concentration

The cytokine concentrations of IL-1ß, MCP-1, TNF-α, MCP-1, IL-4, IL-5, IL-10, CXCL5 and RANTES in the conditioned media were measured by Luminex performance assays (R&D Systems Inc.). Plates were read using a Luminex 100 analyzer (PerkinElmer Life Inc.,) and xPONENT software (Luminex). The concentrations of IL-1ß varied from 5.15 to 528.39 pg/ml, those of TNF-α from 7.76 to 133.68 pg/ml, MCP-1 from 7.28 to 111.16 ng/ml, RANTES from 2266.1 to 10,221.4 pg/ml, IL-10 from 4.43 to 60.0 pg/ml, IL-4 from 5.12 to 17.0 pg/ml, IL-5 from 2.31 to 2.69 pg/ml and those of CXCL5 from 159.52 to 6579.96 pg/ml.

C-reactive protein (hs-CRP) was measured by a high-sensitivity immunoturbidimetric assay method (Cobas Mira Plus, Roche).

### Gene expression in activated endothelial cells

Total RNA was isolated from HUVECs treated with 5% ATCM or SVFCM in EBM-2 with 2% fetal bovine serum in parallel-run samples. Samples of the experiment with the addition of cytokine inhibitors (IL-1β, TNF-α, and MCP-1) to ATCM stimulation were examined in a separate run. The RNeasy Plus mini kit (Qiagen) was used for RNA isolation. A total of 300 ng isolated RNA was used for reverse transcription. After DNase I (Sigma Aldrich) treatment to eliminate DNA from the RNA preparations, cDNA was generated according to the manufacturer’s instructions using the High Capacity RNA-to-cDNA Master Mix Kit (Life Technologies). Levels of gene expression of the cytokines and adipokines of interest were subsequently determined on the Corbett Life Science Rotor Gene 3000 system (Qiagen) using the 5x HOT FIREPol® EvaGreen® qPCR Mix Plus (Solise BioDyne). The nucleotide sequences of the primer pairs of MCP-1 (CCL2, C-C motif chemokine ligand 2, forward 5ʹ-agaagctgtgatcttcaagacc-3ʹ, reverse 5ʹ-agctgcagattcttgggttg-3ʹ), IL-6 (forward 5ʹ-agcagcaaagaggcactggca-3ʹ, reverse 5ʹ-tgaggaacaagccagagctgtgc-3ʹ), nitric oxide synthase 3 (NOS3, forward 5′-gtggctgtctgcatggacct-3′, reverse 5′-ccacgatggtgactttggct-3′), transforming growth factor beta 1 (TGF-β, forward 5′-tcgccagagtggttatctttt-3′, reverse 5′-tagtgaacccgttgatgtcc-3′), selectin E (SelE, forward 5ʹ-tcacagtgtttcgacagctg-3ʹ, reverse 5ʹ-gcaaatgcatggagggttgt-3ʹ), intercellular adhesion molecule 1 (ICAM-1, forward 5ʹ-tcttcctcggccttcccata-3ʹ, reverse 5ʹ-catttggggccatggtacct-3ʹ), and vascular cell adhesion molecule 1 (VCAM-1, forward 5ʹ-cctggaggaaggcagttctg-3ʹ, reverse 5ʹ-ccaggctggaagaagcagaa-3ʹ). Glyceraldehyde 3-phosphate dehydrogenase (forward 5′-agggctgcttttaactctggt-3′; reverse 5′-ccccacttgattttggaggga-3′) levels were used as endogenous controls for normalization. Relative gene expression was calculated using the ΔΔCt method [41] and compared to the respective adipose tissue.

## Data analysis

Statistical analyses were performed with Prism biostatistics software, version 5 (GraphPad Prism). Levels of adhesion were compared by one-way ANOVA followed by *post hoc* Bonferroni’s multiple comparison test. Outliers were detected using Dixon’s Q test. The t-test or Wilcoxon sign-ranked repeated measures test were performed for relative gene expression analysis according to the Shapiro-Wilk test for normality. The comparison of paired samples (SVFCM and ATCM) was analyzed by the paired t-test. Linear regression analysis was used for correlation relationships. Probability values of less than 0.05 were considered significant.
